# Effects of dog cardiac orientation on vertebral heart score measurements in different thoracic types

**DOI:** 10.14202/vetworld.2024.2635-2643

**Published:** 2024-11-25

**Authors:** Suphat Soeratanapant, Somchin Sutthigran, Phasamon Saisawart, Nardtiwa Chaivoravitsakul, Kongthit Horoongruang, Luksamee Limpongsai, Artima Tantarawanich, Chutimon Thanaboonnipat, Kittipong Tachampa, Nan Choisunirachon

**Affiliations:** 1 Department of Surgery, Faculty of Veterinary Science, Chulalongkorn University, Bangkok, Thailand; 2 The Small Animal Hospital, Faculty of Veterinary Science, Small Animal Teaching Hospital, Chulalongkorn University, Bangkok, Thailand; 3 Department of Physiology, Faculty of Veterinary Science, Chulalongkorn University, Bangkok, Thailand

**Keywords:** cardiac dimension, computed tomography, dog, heart orientation, thoracic cage

## Abstract

**Background and Aim:**

Computed tomographic (CT) images can elucidate the variations of cardiac orientation that this information among dog breeds has never been reported. This study aimed to explore the heart orientations of dogs with different thoracic types and study their effects on vertebral heart score (VHS) measurements using CT images.

**Materials and Methods:**

Thoracic CT images of 115 mature dogs without thoracic abnormalities were retrospectively examined. The dogs were classified into four groups: Normal Broad, Abnormal Broad, Normal, and Deep. All dogs were also classified based on their heart deviations. The VHSs were evaluated using lateral VHS, dorsal VHS, and adjusted VHS, and all were compared.

**Results:**

In the normal broad and abnormal broad groups, the lateral VHS and lateral long-axis dimensions were significantly lower than those obtained from the dorsal and adjusted VHSs. In addition, heart deviations were mostly observed in the normal broad and abnormal broad groups. Nevertheless, little evidence was found in the normal and deep groups. The lateral VHS and lateral long-axis dimensions were significantly reduced by heart deviation more than the dorsal and adjusted VHSs.

**Conclusion:**

Cardiac orientations among dog breeds can affect VHSs of lateral projection, especially in the broad thoracic group. Clinical evaluation of the VHS in the broad thoracic dogs should be performed on the dorsal view for more accurate measurement of heart size.

## Introduction

Heart disease is a common dog disease that accounts for 10% of all hospital visits [1]. Heart disease is mostly observed in geriatric dogs [2]. Progression of heart disease results in congestive heart failure, which increases the mortality rate [3]. Therefore, early diagnosis and treatment of heart disease can delay disease progression and prolong lifespan [4]. Although echocardiography is the gold standard for diagnosing heart diseases, it requires user skills and experience [1]. In contrast, thoracic radiography, a diagnostic imaging tool, aids in the detection of heart enlargement and pulmonary edema [4].

Thoracic radiographs are commonly used to estimate heart size and to indicate cardiomegaly. The vertebral heart score (VHS) is the most commonly used technique for evaluating heart size [5]. VHS is a good predictive tool for cardiomegaly due to its moderate correlation with echocardiographic findings [6]. Although the reference value of dog VHS is 9.7 ± 0.5 vertebral units (V) [5], the cutoff value of the VHS device in the case of heart remodeling-induced cardiomegaly is 10.5 V [1, 5]. However, the VHS patterns vary among dog breeds. The normal VHS values of Beagle [7], Boston Terrier [8], Boxer [9], Cavalier King Charles Spaniel [9], Doberman [9], German Shepherd [9], English Bulldog [8], Labrador Retriever [9], Norwich Terriers [10], and Pug [8, 11] were greater than the dog referent value [12]. In addition to breed-related factors that induce VHS values, radiographic positioning may affect VHS. Despite being less clinically significant, the same positioning on thoracic radiography should be used for heart size monitoring [13, 14]. The radiographic positioning and breed variations that affect VHS are believed to be related to the superimposition of a radiographic image. The shape of the dog thorax is a structural variation that may affect the VHS value of breeds [5]. This may be due to the effect of different heart alignments on the cardiac silhouette. To determine this, a correlation analysis between the VHS and the thoracic conformation observed through the thoracic depth (TD) to thoracic width (TW) ratio (TD/TW) was performed [10, 15]. However, these studies did not find a significant correlation between VHS and TD/TW. This might be due to the limited number and variety of dogs included in these studies. In addition, only two-dimensional superimposition images obtained through radiography were used for the evaluation in these studies. It is possible that the heart axis on the lateral thoracic radiograph may not be the true heart axis because the heart measurement in a two-dimensional image cannot define the actual alignment or its orientation.

Computed tomography (CT) is an advanced diagnostic tool that has been increasingly used to further investigate companion animals. CT can provide multiplane images with more details, thereby facilitating more accurate diagnosis [16–18]. Compared with thoracic radiographs, which have less contrast resolution and show superimposition [19], cross-sectional CT images provide an excellent view of the intra-thoracic organs of the patient, including their position, alignment, shape, and size [19, 20]. Despite the similar thoracic cages among people, the human heart can vary in its alignment, and CT images can be used to assess human heart alignment and its actual position [21]. Therefore, different heart alignments may be affected in dogs with different thoracic conformations. Different heart orientations can affect the two-dimensional cardiac silhouette and its measurement, such as the VHS evaluation performed on radiographs.

To the best of our knowledge, no information on the orientation of the heart in dog breeds with different thoracic types has been reported. This crucial information may affect the heart size assessment using VHS. Therefore, this study aimed to determine dog heart orientation in dogs with different thoracic types and evaluate the effect of heart orientation on VHS evaluation using CT images. The present study hypothesized that dog thoracic conformation affects heart orientation. In addition, various heart orientation effects on the actual heart dimension were evaluated using the VHS.

## Materials and Methods

### Ethical approval

All recorded data, including signalment, history, and images, were approved by the committee of the Small Animal Hospital, Faculty of Veterinary Science, Chulalongkorn University (S448/2566).

### Study period and location

This study was conducted from August 2023 to March 2024 using the retrospective data of dogs who underwent CT examination at the Small Animal Hospital, Faculty of Veterinary Science, Chulalongkorn University.

### Inclusion criteria

CT images from the Diagnostic Imaging Unit of The Small Animal Hospital, Faculty of Veterinary Science, Chulalongkorn University, fitting the inclusion criteria, were included in the study. The inclusion criteria were good quality non-contrasted enhancement, thoracic CT images of dogs >1 year of age, mutuality of the vertebral columns, and maximal slice thickness of the CT images (1.25 mm). In addition, dogs with minimal vertebral malformation, such as hemivertebra, or spine degeneration, such as spondylosis, were also included.

### Exclusion criteria

Dogs altered with thoracic abnormalities that disturb vision of the thoracic cavity, such as pleural disease, mediastinal disease, or thoracic wall diseases; dogs with a history of heart disease, such as cardiomegaly or dextrocardia; dogs with lung atelectasis or lung collapse; and dogs with abnormal alignment of vertebrae that interfered with the VHS evaluation, such as kyphosis, including dogs with abnormal sternum, such as pectus excavatum were excluded from the study.

### CT image acquisition

The included CT images of all dogs were acquired under general anesthesia with chemical anesthetic agents for premedication and induction, which followed the dog’s condition and anesthesiologist preferences. The dogs were maintained for the anesthetic stage with isoflurane. All dogs were scanned using a 64-slice, helical CT scanner (Optima CT660, GE Healthcare, Tokyo, Japan) and were positioned on the sternal recumbency plane.

### Experimental protocols

**Figure-1:**
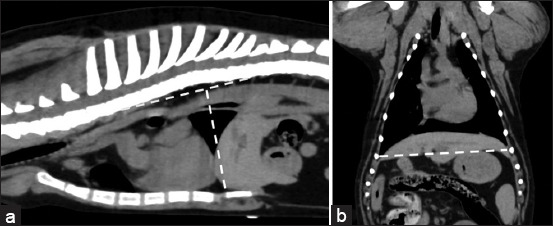
Soft-tissue window of pre-contrast enhancement. (a) Sagittal and (b) dorsal computed tomographic images of dog thorax show the technique used to measure (a) thoracic depth and (b) thoracic width.

**Figure-2:**
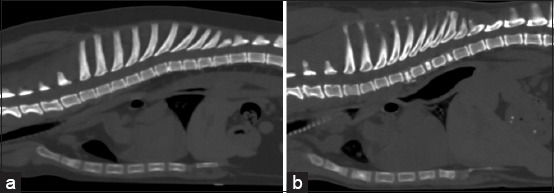
Bone window of pre-contrast enhancement. The sagittal computed tomographic images of the dog thorax show the characteristics of the thoracic vertebrae in broad thoracic dogs, which are (a) normal vertebrae and (b) abnormal vertebrae.

CT images were collected as DICOM files and reanalyzed using DICOM viewer software (OsiriX^®^DICOM viewer software). To evaluate the thoracic structures, multiplanar reconstruction (MPR) was applied to all CT images, and a soft-tissue window (350 Hounsfield units [HU] of window width [WW] and 40 HU of window level [WL]) was established during soft-tissue assessment. All dogs were primarily assessed for TD and TW to classify the thoracic conformation type, which was modified from a previous report using radiographs [5]. TD was measured in a sagittal plane along a line perpendicular to the vertebral columns as a line from the cranial edge of the xiphoid process to the ventral border of the vertebral column. The TW was measured on the dorsal plane, and a line was drawn as the distance between the medial borders of the eighth ribs at their greatest dimension (Figure-1). Then, the TD/TW ratio was calculated. Dogs were classified into broad thorax (Broad) if TD/TW was <0.75, deep thorax (Deep) if TD/TW was >1.25, and normal thorax (Normal) if TD/TW was between 0.75 and 1.25 (Normal) [5]. In the broad group, dogs were further classified into two subgroups depending on their vertebral characteristics: Broad with normal vertebrae (Normal Broad) and broad with abnormal vertebrae (Abnormal Broad) groups (Figure-2).

**Figure-3:**
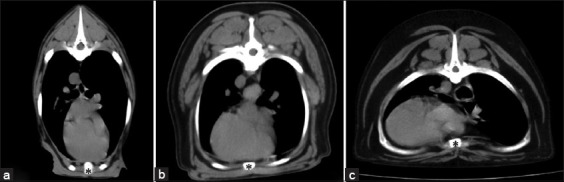
Soft-tissue window of pre-contrast enhancement, transverse computed tomographic images of the dog thorax show the location of the cardiac apex that were categorized as 0, –1, and –2. (a) The heart axes were recorded as 0 if a position of the heart apex was on the sternum (asterisk), (b) the heart apex was recorded as –1 if a position of the heart apex diverted from the sternum (asterisk) but not diverted over the costochondral junction, and (c) the heart apex was recorded as –2 if a position of the heart apex diverted from the sternum (asterisk) and over the costochondral junction.

**Figure-4:**
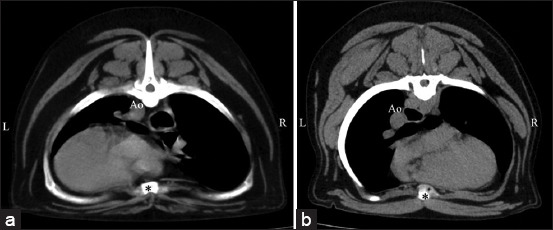
Soft-tissue window of pre-contrast enhancement, transverse computed tomographic images of the dog thorax showing grade 2 of heart deviation into either (a) the left side or (b) the right side compared with the sternum (asterisks). Ao indicates the descending aorta.

The heart alignment was observed in a cross-sectional plane, including its orientation as the location and direction of the heart apex. Heart alignment was designated as grade 0 if the position of the heart apex was on the sternum, grade 1 if the heart apex diverted from the sternum but did not divert over the costochondral junction, and grade 2 if the heart apex diverted from the sternum and over the costochondral junction (Figure-3). In addition, the direction of the heart apex deviation, either to the right or left hemithorax, in all dogs was noted (Figure-4).

**Figure-5:**
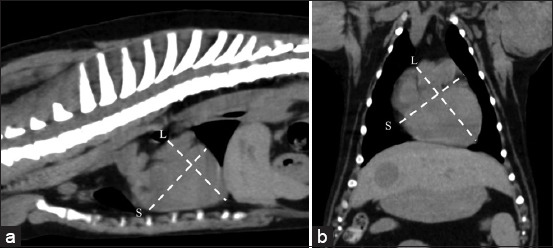
Soft tissue of pre-contrast enhancement. (a) Sagittal and (b) dorsal computed tomographic images of dog thorax show the technique used to measure (a) the lateral cardiac long (L) and short (S) cardiac axes and (b) the dorsal long (L) and short (S) cardiac axes.

**Figure-6:**
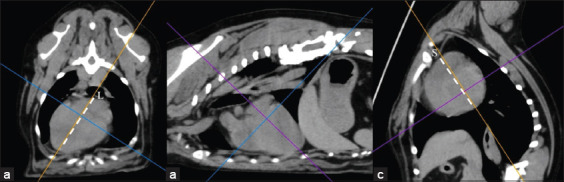
(a-c) Soft-tissue window of pre-contrast enhancement. The multiplanar reconstruction computed tomographic images of dog thorax show the technique used to measure the adjusted cardiac long (L) and short (S) axes on thoracic computed tomography images.

Subsequently, VHS was evaluated in the sagittal plane, which is a technique similar to that is indicated for lateral VHS [5]. Briefly, the long-axis dimension was measured from the carina to the apex of the heart or at the sternum. The short-axis dimension was measured at the junction of the caudal aspect of the cardiac silhouette and the ventral border of the caudal vena cava [5], and measurements were performed at a 90° angle perpendicular to the long-axis dimension (Figure-5a). Moreover, the long- and short-axis dimensions on the dorsal plane were measured, and these lines were also normalized to the thoracic vertebrae on the sagittal plane; the VHS was recorded as the dorsal VHS [22] (Figure-5b). In addition, the maximal dimensions of the heart, both long- and short-axis dimensions, were adjusted using the MPR to achieve maximal alignment of the heart among dogs. In the MPR image, the adjusted long-axis dimension was defined as the line from the carina to the tip of the left ventricle. The short-axis dimension was the line from the widest edge from the cranial to the caudal side of the heart on the dorsal plane, which is related to the lateral short-axis dimension and located on the sagittal plane (Figure-6). Subsequently, the heart’s long- and short-axis dimensions on the adjusted plane were transposed and measured at the thoracic vertebrae in the sagittal plane. The results indicate adjusted VHS. CT images were acquired by two operators, a well-trained graduate student and a radiologist with more than 20 years of experience in the clinical field.

All lateral, dorsal, and adjusted VHSs in both the long- and short-axis dimensions were normalized to the thoracic vertebrae on the bone window (1,500 HU of WW and 300 HU of WL) on the sagittal image. The long- and short-axis dimensions were neutralized with the thoracic vertebrae starting from T4 and recorded as the number of the vertebrae; V [12].

### Statistical analysis

GraphPad Prism 7 software (GraphPad Software, CA, USA) was used for statistical analysis. The Shapiro–Wilk test was used to test the data distribution. All clinical and demographic data were described as descriptive analyses. According to data distribution, normally distributed data are presented as mean and standard deviation, whereas non-normally distributed data are presented as median, minimum, maximum, and range. The comparison between lateral, dorsal, and adjusted VHSs, including the long- and the short- axis dimensions from each technique on thoracic CT images among dogs with different thoracic types, was tested by analysis of variance or Kruskal–Wallis’s test, depending on data distribution, followed by Tukey’s test. In addition, the relative association between thoracic conformation and heart orientation was estimated using odds ratios (ORs) and 95% confidence intervals (CIs). The intraclass correlation coefficient was used to evaluate interobserver agreement. p < 0.05 was considered statistically significant.

## Results

### Clinical and demographic data

A total of 115 CT images of dogs that fit the inclusion criteria were included in this study. All statistical data, such as the number of dogs in each group, their ages, body weights, and sex, and the number of dogs in each sex, are reported in Table-1. In the normal broad group, there were Golden retriever (n = 7; 17.5%), three dogs (7.5% each) from each breed of Labrador retriever and poodle, two dogs (5.0% each) from each breed of Cocker Spaniel, German Shepperd, Rottweiler, Shih Tzu, Pug, Pekingese, Yorkshire Terrier, and a dog (2.5% each) from each breed of Bang Kaew, Beagle, Chihuahua, Cavalier King Charles Spaniel, Corgi, Jack Russell, Maltese, Pit Bull, Pomeranian, Schnauzer, Scottish terrier, and Splitz. In the abnormal broad group, there were 22 French Bulldogs (n = 22; 88%), two Bulldogs (n = 2; 8%), and one Pug (n = 1; 4%). In the normal group, there were mixed breeds (n = 12; 27.2%), German Shepherds (n = 5; 11.3%), Thai Ridgeback (n = 3; 6.8%), and two dogs (4.5% each) from the Bang Kaew, Jack Russell, Poodle, Siberian Husky, and Yorkshire Terrier breeds, and one dog (2.2% each) from each breed of Fox Terrier, Labrador Retriever, Pomeranian, Australian Cattle dog, Beagle, Bernese Mountain dog, Boxer, Bullmastiff, Collie, Cocker Spaniel, Corgi, Chihuahua, Dachshund, and Spitz. The deep group included mixed breed (n = 3; 50%), German Shepherd (n = 2; 33.3%), and Italian Greyhound (n = 1; 16.7%).

**Table-1:** Clinical demographic data including age, body weight, and number of dogs of each sex in all dogs and dogs in each thoracic group.

Parameters	Overall (n = 115)	Normal broad (n = 40)	Abnormal broad (n = 25)	Normal (n = 44)	Deep (n = 6)
Age (years)	7.43 ± 0.38	7.42 ± 0.74	6.11 ± 0.59	8.50 ± 0.60	6.63 ± 3.00
	7.50	7.00	5.30	10.00	2.90
	(1.00-21.00)	(1.00-21.00)	(1.00–12.00)	(1.00-17.00)	(1.00–17.00)
Body weight (kg)	17.58 ± 0.99 14.80	17.95 ± 2.89	14.37 ± 0.84 14.15	17.90 ± 1.59	16.50 ± 3.60
	(1.00–48.00)	10.00	(7.00–17.00)	17.70	15.50
		(1.00-47)		(2.50–48.00)	(4.30 –26.90)
Males	71 (63.4%)	22 (55.0%)	21 (84.0%)	24 (54.5%)	4 (66.7%)
Females	44 (36.5%)	18 (45.0%)	4 (16.0%)	20 (45.5%)	2 (33.3%)

### Location of the heart apex in dogs

**Figure-7:**
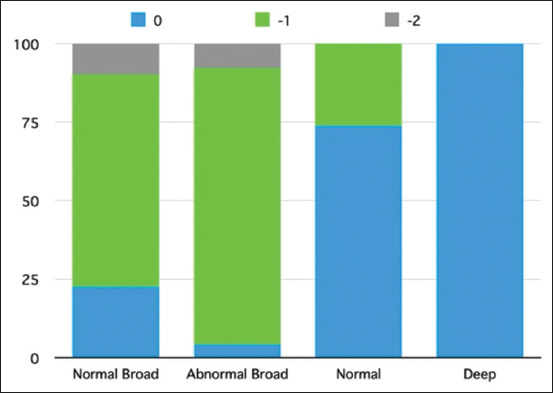
Percentage of dogs among groups of the normal board, abnormal board, normal, and deep groups that had the deviation of the heart apex as grades 0, −1, and −2.

**Figure-8:**
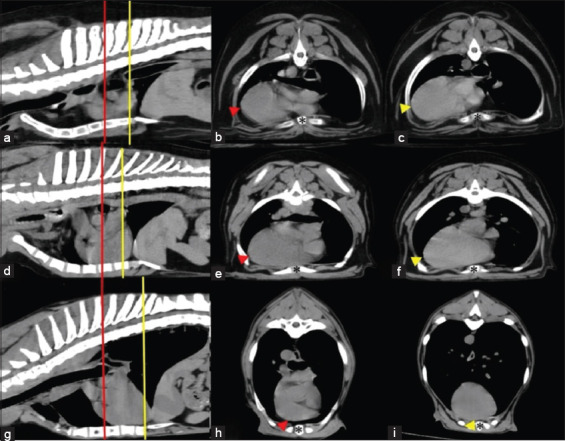
Soft-tissue window of pre-contrast enhancement. The multiplanar reconstruction computed tomographic images of dog thorax with different thoracic types (a–c) are the broad thoracic type, (d–f) are the normal thoracic type, and (g–i) are the deep thoracic type. The red and yellow lines represent the heart orientation at the carina level and the actual apex on the transverse plane. The red and yellow arrowheads indicate the cardiac apex related to the red line and the yellow arrowhead indicates the cardiac apex related to the yellow line. Asterisks indicate sternum.

Table-2, Figures-3 and 4 present information on heart orientation classified as grades 0, 1, and 2, including the deviation of the heart apex in all dogs and dogs in each thoracic group. The broad thoracic dogs, which included the normal and abnormal broad groups, had significantly higher ORs of developing heart deviation (OR, 12.83; 95% CI 5.29–31.73; p < 0.0001) than the non-broad thoracic dogs, which included the normal and deep groups (Figures-7 and 8).

**Table-2:** The distribution of heart apex orientation in all dogs and dogs in each thoracic group.

Heart apex deviation	Overall (n = 115) (%)	Normal broad (n = 40) (%)	Abnormal broad (n = 25) (%)	Normal (n = 44) (%)	Deep (n = 6) (%)
0	45 (39.1)	9 (22.5)	1 (4.0)	29 (65.9)	6 (100.0)
–1	64 (55.6)	27 (67.5)	22 (88.0)	15 (34.1)	0 (0.0)
Right	8 (6.9)	2 (5.0)	2 (8.0)	4 (9.1)
Left	56 (48.6)	25 (62.5)	20 (80.0)	11 (25.0)
–2	6 (5.2)	4 (10.0)	2 (8.0)	0 (0.0)	0 (0.0)
Right	1 (0.8)	1 (2.5)	0 (0.0)	
Left	5 (4.3)	3 (7.5)	2 (8.0)	

0: Grade 0 if the position of the heart apex was on the sternum. –1: Grade 1 in which the position of the heart apex diverted from the sternum but did not divert over the costochondral junction. –2: Grade -2 wherein the position of the heart apex diverted from the sternum and over the costochondral junction

### Assessment of heart size using VHS based on technique and degree of heart orientation in all thoracic types of dogs

First, two operators evaluated the interobserver reliability. The intraclass correlation coefficients of the lateral and adjusted VHS measurements showed good reliability (r = 0.81 for both lateral and adjusted VHS measurements), whereas the dorsal VHS measurement showed excellent reliability (r = 0.93).

The VHS information, which included the long-axis dimension, the short-axis dimension, and the total VHS for each technique of the lateral VHS, the dorsal VHS, and the adjusted VHS, is reported in Table-3. The short-axis dimensions were not significantly different among the lateral, dorsal, and adjusted VHS techniques in all thoracic groups (normal broad, p = 0.98; abnormal broad, p = 0.45; normal thorax, p = 0.08; and deep thorax, p = 0.58). Notably, the long-axis dimensions of the normal and abnormal broad thorax were significantly lower in the lateral VHS group than in the dorsal and adjusted VHS groups (p < 0.0001 for all comparisons), while the long-axis dimensions of the dorsal and adjusted VHS groups were not significantly different (p = 0.38 and p = 0.58 for the normal broad and abnormal broad thorax, respectively). In the normal and deep groups, the long-axis dimensions showed no significant differences among the lateral, dorsal, and adjusted VHSs (p = 0.07 and p = 0.12 for the normal and deep groups, respectively). In summary, significant differences between lateral and dorsal VHSs and between lateral and adjusted VHSs were detected in the normal broad group (p < 0.0001 for both comparisons). However, there were no significant differences in the dorsal and adjusted VHSs (p = 0.58). Similar results were found in the abnormal broad group; there were significant differences between the lateral and dorsal VHSs (p < 0.001) and lateral and adjusted VHSs (p = 0.019). However, no significant difference was observed between the dorsal and adjusted VHSs (p = 0.36). In the normal and deep groups, no significant differences were observed among the lateral, dorsal, and adjusted VHSs (p = 0.22 and p = 0.20 for the normal and deep groups, respectively).

**Table-3:** Summary of VHS parameters including the short and long axes, and total VHS (total) in each thoracic group of dogs.

VHS parameters (V)	Normal broad (n = 40)	Abnormal broad (n = 25)	Normal (n = 44)	Deep (n = 6)
Lateral				
Short-axis	4.38 ± 0.06 (3.20–5.30)	5.76 ± 0.17 (4.20–7.10)	4.26 ± 0.06 (3.30–5.10)	4.05 ± 0.09 (3.80–4.10)
Long-axis	4.38 ± 0.09^a^ (2.90–5.60)	5.54 ± 0.18^a^ (3.90–7.00)	4.92 ± 0.08 (3.80–6.10)	4.80 ± 0.36 (4.60–5.50)
Total	8.76 ± 0.13^b^ (7.10–10.90)	11.31 ± 0.33^b^ (8.30–14.00)	9.17 ± 0.12 (7.70–11.20)	8.85 ± 0.43 (6.90–9.80)
Dorsal				
Short-axis	5.39 ± 0.08 (4.20–6.80)	6.08 ± 0.18 (4.30–7.90)	4.14 ± 0.05 (3.60–4.80)	3.43 ± 0.38 (2.00–4.70)
Long-axis	5.57 ± 0.11 (4.20–7.50)	7.08 ± 0.15 (5.50–8.30)	5.10 ± 0.08 (4.10–6.00)	4.43 ± 0.18 (4.50–5.50)
Total	9.96 ± 0.17 (7.90–12.50)	13.29 ± 0.33 (9.80–16.20)	9.30 ± 0.13 (7.60–11.00)	7.86 ± 0.54 (6.10–9.50)
Adjusted				
Short-axis	4.39 ± 0.07 (3.20–5.70)	5.86 ± 0.19 (4.20–8.20)	4.27 ± 0.06 (3.50–4.90)	3.88 ± 0.20 (3.90–4.20)
Long-axis	5.39 ± 0.08 (4.20–6.80)	6.62 ± 0.18 (5.00–8.40)	5.20 ± 0.07 (4.30–6.20)	5.05 ± 0.14 (4.50–5.50)
Total	9.75 ± 0.13 (8.20–11.60)	12.64 ± 0.34) (9.80–15.60)	9.30 ± 0.13 (7.60–11.00)	8.93 ± 0.32 (8.90–9.60)

Data are presented as mean ± standard deviation including minimal–maximal value.

a Significantly lower compared to the adjusted long-axis and dorsal long-axis VHS in the same group.

b Significantly lower than the adjusted total VHS and dorsal total axis VHS groups. VHS: Vertebral heart score

In 115 dogs (Table-2), the short- and long-axis dimensions, including the VHS of dogs with grade 0 heart deviation, were not significantly different between the lateral, dorsal, and adjusted VHSs (p = 0.07, p = 0.11, and p = 0.21 for the short-axis dimension, the long-axis dimension, and VHS, respectively). In grade 1, the short-axis dimension was not significantly different between the lateral, adjusted, and dorsal VHSs (p = 0.95). In contrast, the long-axis dimension was significantly lower in the lateral VHS group than in the dorsal and adjusted VHS groups (p < 0.0001 for both comparisons), whereas the long-axis dimensions of the dorsal and adjusted VHS groups were not significantly different (p = 0.60). Likewise, the lateral VHS was significantly lower than the dorsal and adjusted VHSs (p = 0.002 for the lateral versus dorsal VHS and p = 0.02 for the lateral versus the adjusted VHS values), whereas the dorsal and adjusted VHSs were not significantly different (p = 0.79). Similar results were found for grade 2 heart deviations. The short-axis dimension was not significantly different between the VHS techniques (p = 0.69). However, the long-axis dimensions of the lateral VHS were significantly lower than those of the dorsal and adjusted VHSs (p < 0.0001 and p = 0.003, respectively), whereas the long-axis dimensions of the dorsal and adjusted VHSs were not significantly different (p = 0.10). Likewise, the lateral VHS was significantly lower than the dorsal and adjusted VHSs (p = 0.004 and p < 0.0001 for lateral VHS versus the dorsal and lateral VHS and Adjusted VHS, respectively), whereas the dorsal and adjusted VHSs were not significantly different (p = 0.39) (Table-4).

**Table-4:** Summary of VHS parameters for each heart apex deviation group in all dogs.

VHS parameters (V)	0 (n = 45)	–1 (n = 64)	–2 (n = 6)
Lateral			
Short-axis	4.30 ± 0.05 (3.30–5.30)	4.85 ± 0.11 (3.20–7.10)	4.60 ± 0.30 (3.90–5.90)
Long-axis	4.97 ± 0.08 (3.80–6.10)	4.89 ± 0.11^a^ (3.50–7.00)	3.80 ± 0.35^a^ (2.90–5.00)
Total	9.28 ± 0.12 (6.90–10.90)	9.74 ± 0.22^b^ (7.20–14.00)	8.40 ± 0.57^b^ (7.10–10.90)
Adjusted			
Short-axis	4.24 ± 0.07 (3.80–4.50)	4.90 ± 0.12 (3.20–7.10)	4.86 ± 0.31 (4.20–6.20)
Long-axis	5.25 ± 0.07 (4.40–6.20)	5.77 ± 0.12 (4.20–8.40)	5.78 ± 0.25 (5.00–6.50)
Total	9.50 ± 0.13 (7.40–11.60)	10.67 ± 0.23 (8.00–15.60)	10.65 ± 0.54 (9.30–12.70)
Dorsal			
Short-axis	4.10 ± 0.09 (3.20–5.00)	4.95 ± 0.13 (3.10–7.90)	5.01 ± 0.40 (4.10–6.80)
Long-axis	5.12 ± 0.08 (4.00–6.20)	5.94 ± 0.13 (4.10–8.30)	6.86 ± 0.41 (5.50–8.20)
Total	9.10 ± 0.17 (7.30–11.00)	10.89 ± 0.26 (7.90–16.20)	11.88 ± 0.79 (9.80–15.00)

Data are presented as mean ± standard deviation including minimal–maximal value.

a Significantly lower than the adjusted long-axis and dorsal long-axis VHSs in the column.

b Significantly lower than the adjusted total VHS and dorsal total axis VHS in the column. VHS: Vertebral heart score

## Discussion

The VHS score has been accepted as a radiographic score useful for evaluating dogs’ heart size [1]. The reference value of dog VHS 9.7 ± 0.5 V [12] could not be applied to each dog because the VHS values of most breeds differed from the reference value [7–11]. In addition, radiographic positioning can also affect VHS. A previous report found that right lateral recumbency had a higher VHS value than left recumbency [13, 14]. The different VHSs due to radiographic positioning and breed variations are believed to be related to the superimposition effect of radiographic images, which may be related to the shape of the thoracic conformation in dogs [11]. However, there are no reports on the thoracic conformation of dogs that affect heart orientation and VHS values. Therefore, this study explored the variation in heart orientations among dogs with different thoracic types using thoracic CT images, which can provide information about the thoracic structures without superimposition [23, 24]. In addition, this study explained the variabilities of the VHS, composed of short-axis, long-axis, and total dimensions, and compared these values among the lateral, adjusted, and dorsal VHSs in dogs with different thoracic types to obtain more information on the heart orientation that may affect the VHS scoring procedure.

Although lateral VHS following Buchanan VHS has been widely used on thoracic radiographs [1, 12], adjusted VHS can evaluate the actual heart dimension without superposition by applying MPR. In addition, the dorsal VHS represents a VHS measured on the dorsal plane that is similar to ventrodorsal (VD) thoracic radiographs [12]. The results from these parameters among different dog breeds showed that the lateral VHS of the broad groups (normal broad and abnormal broad) were significantly lower than those obtained from the adjusted and dorsal groups. The long-axis dimension was the main parameter affecting the total VHS in the broad groups. The MPR images showed that the heart apex of most dogs in these groups deviated from the sternum. This might be a major factor that affects the long-axis dimension of a lateral projection due to the foreshortening effect, in which the long-axis dimension is not aligned parallel to the image detector [12]. The effect of heart deviation in the broad groups might be due to the relatively short dimensions of the TD. Notably, the dorsal VHS was not significantly different from the adjusted VHS value. This may be due to the lateral deviation of the heart axis in the broad groups. The heart orientations of these dogs were expanded, particularly in the long-axis dimension of the dorsal plane. Therefore, the long-axis dimension of the broad groups in this view was similar to the adjusted heart axes and provided more accurate results compared with the lateral VHS obtained from the lateral view. This could be an explanation for the previous study observed by Tangpakornsak *et al*. [25] in Corgi dogs, which found that VHS on a VD radiograph was greater than VHS on a lateral view because the measurement of the long-axis dimension was evaluated as the heart dimension relative to the actual heart apex on the VD view.

There were no significant differences between the deep and normal thoracic groups and between the lateral, adjusted, and dorsal VHSs. These results support the hypothesis that the thoracic conformation affects the heart orientation. The deep and normal groups had a higher TD/TW, which narrowed the thorax and was higher than that of the broad group. This type of thorax, a long-axis dimension of the heart, can align vertically and show the actual long-axis dimension on the laterolateral radiographic image due to the lesser foreshortening effect than the broad groups. In addition, the heart deviation in most dogs in the deep and normal thorax groups was grade 0. The results showed that grade 0 of the heart orientation did not significantly interfere with the VHS values.

Although a previous study by Bodh *et al*. [15] found that TD/TW did not affect VHS, the breeds included in these studies had a narrow range of TD/TW. The breeds used in one study were Spitz, Labrador Retriever, and Mongrel dogs. These dogs had means of TD/TW of 0.82 ± 0.05, 0.89 ± 0.04, and 0.93 ± 0.01, respectively. Jepsen-Grant *et al*. [8] reported that Pug, Pomeranian, Yorkshire Terrier, Dachshund, Bulldog, Shih Tzu, Lhasa Apso, and Boston Terrier had mean TD/TW of 0.75 ± 0.8, 0.75 ± 0.08, 0.70 ± 0.07, 0.83 ± 0.07, 0.77 ± 0.10, 0.79 ± 0.06, 0.80 ± 0.08, and 0.78 ± 0.07, respectively. The present study included a greater variety of dogs of each thoracic type. Therefore, the current result better explains the effect of different TD/TWs of different thoracic types on the heart orientation and VHS value.

Notably, the German Shepherd has been classified as having a deep thorax; however, the results of this study showed that the TD/TW was <0.75 and the dog was classified as the broad group [26, 27]. In addition, although Pomeranians have been reported to be of the normal thoracic type [8], some Pomeranians in the present study belonged to both the normal broad and normal groups. The effect of heart deviation in some dogs might be due to the relatively short dimension of the TD, which relates to the flat ventral thoracic wall caused by the flat-to-concave alignment of the chondral rib. Therefore, breed-specific thoracic conformation could not be assessed in all dogs in the present study. We suggest that TD/TW should be evaluated primarily to classify the thoracic type of dogs before heart size measurements.

The direction of the heart apex illustrated that most dogs had heart deviations into the left hemithorax; however, some dogs had heart deviations into the right side (Figure-4). All dogs with the right side deviation in this study were defined as dextroposition. The heart deviated to the right from the extracardiac cause, such as a thoracic conformation without situs inversus different from the term dextrocardia, which is the right-sided embryological development of the heart [28]. Due to the smaller number of right heart deviations, side deviation did not affect the VHS value, which was not observed. Likewise, the degree of the heart deviation significantly influenced the long-axis dimension and the total VHS number when comparing the lateral, dorsal, and adjusted groups. However, this difference was not found in the grade 0 heart orientation. These results showed that heart deviation affects the VHS, especially the lateral VHS. However, the number of dogs with grade-2 heart deviations was small. Further studies are needed to explore the correlation between the degree of heart deviation and the length of the long-axis dimension of the heart.

The present study has some limitations. First, due to the study’s retrospective nature, the number of dogs in each group could not be obtained equally due to the different popular breeds. In addition, the dogs were positioned on the sternal recumbency during CT imaging. This positioning is not similar to lateral recumbency in thoracic radiography. The grant of the heart apex at each recumbency may also interfere with the results. Therefore, evaluating cardiac alignment in different positions, such as lateral or dorsal recumbences, observed on CT images, would be more elucidated.

## Conclusion

The VHS in the normal and deep thoracic group that had a TD/TW >0.75 presented the actual heart size measured on lateral projection, while the VHS of the broad group, including the abnormal and normal broad groups, could be lesser than the actual heart size due to the foreshortening effect of the long-axis dimension. Therefore, the VHS data in the broad thorax group should be measured in the dorsal view because it provides a more accurate measurement of the heart size that is almost equal to the actual heart size.

## Authors’ Contributions

NC2, KT, CT, and SS1: Conceptualized and designed the study. SS2, PS, NC1, KH, LL, and AT: Performed data collection as per inclusion criteria. NC2, KT, and SS1: Data validation and statistical analysis. NC2 and SS1: Drafted the manuscript. NC2, KT, and CT: Supervised the data collection and analysis. All authors have read and approved the final version.
